# Hospital Annual Meetings

**Published:** 1920-04-24

**Authors:** 


### Hospital Annual Meetings.

### City of London Hospital for Diseases of the Chest.

Presiding over the annual Court of Governors of the City of London Hospital for Diseases of the Chest, held at the Vintners' Hall, Upper Thames Street, last week, Sir A. Kaye Butterworth intimated that although the financial position of the institution at the end of 1919 might be considered satisfactory, the outlook for this year was by no means free from anxiety, especially in view of the urgent need of structural improvements, which, owing to high prices, would, it was estimated, cost £30,000. Expenditure since 1914 had doubled, and would, it was feared, continue to increase. The number of sufferers from consumption was now more owing to the war, and the need of combating the disease, therefore, greater than ever. With a view to raising the considerable sum of money required, the committee has been given power to make reasonable charges to patients. In-patients last year numbered 889, the average in residence daily being 163. Nearly 9,300 new out-patients, whose attendances totalled over 33,600, were also cared for.

### The Infants' Hospital.

Lord Cheylesmore presided last week over the annual Court of Governors of the Infants' Hospital, which in 1919 admitted 266 babies as in-patients. Of that number 120 were discharged as good recoveries, while 104 unfortunately died. New out-patients last year numbered 696, with 2,837 attendances. The total indebtedness of the hospital has been increased to £4,100 by the addition of a deficit of £1,020 during the period under review. The care of infant life being of prime importance to the nation, and at the present time a matter of urgency, the committee trusts that the work of the institute will not be allowed to languish through insufficient financial assistance, and makes an earnest appeal to those who understand the problems involved. With the desire of increasing the usefulness of the hospital the committee is considering proposals for co-operating with public authorities and taking any other steps whereby the equipment of the institution may be utilised to the fullest advantage.

